# β-lactamases *(bla _TEM_, bla _SHV_, bla _CTXM-1_, bla _VEB_, bla _OXA-1_*) and class C β-lactamases gene frequency in *Pseudomonas aeruginosa* isolated from various clinical specimens in Khartoum State, Sudan: a cross sectional study

**DOI:** 10.12688/f1000research.24818.3

**Published:** 2021-04-01

**Authors:** Dina N. Abdelrahman, Aya A. Taha, Mazar M. Dafaallah, Alaa Abdelgafoor Mohammed, Abdel Rahim M. El Hussein, Ahmed I. Hashim, Yousif F. Hamedelnil, Hisham N. Altayb

**Affiliations:** 1 Department of Virology, Central Laboratory, Khartoum, Sudan; 2 Department of Microbiology, College of Medical Laboratory Sciences, Sudan University of Science and Technology, Khartoum, Sudan; 3 Department of Pharmaceutical Biotechnology, College of Pharmacy, Ahfad University for Women, Omdurman, Khartoum, Sudan; 4 Biochemistry Department, Faculty of Sciences, King Abdulaziz University, Jeddah, Saudi Arabia

**Keywords:** ESBLs, class C β-lactamase, Polymerase Chain Reaction, Ps. aeruginosa, pyocyanin pigment, Khartoum-Sudan.

## Abstract

**Background:** *Pseudomonas aeruginosa *is a pathogenic bacterium, causing nosocomial infections with  intrinsic and acquired resistance mechanisms to a large group of antibiotics, including β-lactams. This study aimed to determine the susceptibility pattern to selected antibiotics and to index the first reported β-lactamases genes frequency in *Ps. aeruginosa *in Khartoum State, Sudan.

**Methods: **121 *Ps. aeruginosa *clinical isolates from various clinical specimens were used in this cross sectional study conducted in Khartoum State. Eighty isolates were confirmed as *Ps.* *aeruginosa *through conventional identification methods and species specific primers. The susceptibility pattern of the confirmed isolates to selected antibiotics was done following the Kirby Bauer disk diffusion method. Multiplex PCR was used for detection of seven β-lactamase genes ( *blaTEM, blaSHV, blaCTXM-1, blaVEB, blaOXA-1, blaAmpC and blaDHA*).

**Results: **Of the 80 confirmed *Ps. aeruginosa *isolates, 8 (10%) were resistant to Imipenem while all isolates were resistant to Amoxicillin and Amoxyclav (100%). A total of 43 (54%) *Ps. aeruginosa *isolates were positive for *blaTEM, blaSHV, blaCTXM-1, blaVEB and blaOXA-1* genes, while 27 (34%) were positive for class C β- Lactamases, and 20 (25%) were positive for both classes. Frequency of beta-lactamases genes was as follows: blaTEM, 19 (44.2%); blaSHV, 16 (37.2%); bla CTX-M1, 10 (23.3%); blaVEB, 14 (32.6%); blaOXA-1, 7 (16.3%). blaAmpC 22 (81.5%) and bla DHA 8 (29.6%).  In total, 3 (11.1%) isolates were positive for both bla AmpC and blaDHA genes.

**Conclusion:** *Ps. aeruginosa *isolates showed a high rate of β- lactamases production, with co-resistance to other antibiotic classes. The lowest resistance rate of *Ps. aeruginosa *was to Imipenem followed by Gentamicin and Ciprofloxacin. No statistically significant relationship between production of β-lactamases in *Ps. aeruginosa *and resistance to third generation cephalosporins was found.

## Introduction

*Pseudomonas aeruginosa* is one of the leading causes of nosocomial infections worldwide with high mortality rates, particularly among immunocompromised patients ^1^. *P. aeruginosa* infections are difficult to treat, due to its extraordinary antimicrobial resistance to all available classes of antimicrobial agents ^2^, including β-lactams, aminoglycosides and fluoroquinolones ^3^. Resistance to β-lactams occurs by different mechanisms, including bla _*AmpC*_ overexpression due to genetic mutations, mutant gene acquisition, overproduction of efflux system, or low permeability ^3^. β-lactamases refer to enzymes that hydrolyze the amide bond of the β-lactam ring leading to drug inactivation and therapy failure ^4^. β-lactamases are classified molecularly into four groups: class A (extended spectrum β-lactamases (ESBLs)), class B (metallo-β-lactamases), class C (cephalosporinases), and class D (oxacillinases) ^5^.

ESBLs are enzymes that extend their hydrolyzing ability to hydrolyze broad spectrum cephalosporins ^6^ and they also confer resistance to penicillins and narrow spectrum cephalosporins ^3^. ESBLs are inhibited by β-lactamase inhibitors, such as clavulanic acid ^7^. β-lactamases are transformed to ESBLs usually after point mutations in the β-lactamases gene. These mutations alter the substrate specificity because of changes in the amino acid sequences near the enzyme active site ^5^. ESBLs producing *P. aeruginosa* have been reported worldwide in different countries ^8– 17^.

AmpC β-lactamases are class C cephalosporinases that mediate bacterial resistance to cephalosporins and cephamycins. They also exhibit low rates of monobactam, cefepime and carbapenem hydrolysis ^18^ and usually resist the inhibition by clavulanic acid ^4^. Normally, AmpC is a chromosomal β-lactamase gene that is regulated by ampR gene and expressed constantly. Point mutations of ampR gene in *Enterobacter cloacae* activate AmpC that mediate resistance to β-lactams ^19^. In *P. aeruginosa* over expressed AmpC β-lactamase mediate the resistance to broad spectrum cephalosporins ^3^. AmpC β-lactamase in *P. aeruginosa* has also been reported in different countries around the world ^20– 27^.

The exact frequency of β-lactamase producing *P. aeruginosa* in Khartoum State, Sudan is unknown; therefore, the aim of this study was to determine the susceptibility pattern to selected antibiotics and to determine the frequency of β-lactamases producing *P. areuginosa* isolates collected in Khartoum State hospitals.

## Methods

### Study design

This is a cross-sectional study conducted between February 2017 and October 2017. Ethical approval for the study was obtained from the ethical committee of the College of Medical Laboratory Science, Sudan University of Science and Technology (SUST) (ethical meeting no, SUST/DSR/1EC/EA2/2017; data, 07 ^th^ January 2017). Written informed consent from participants was waived by the same ethical committee as the study only used previously collected human bio-specimens with limited participant data.

### Collection and identification of bacterial strains

A total of 80 *ps. aeruginosa* clinical isolates were obtained from Soba Teaching Hospital, Elribat University Hospital, National Laboratory for Public Health, Ear Nose Throat Hospital, and Military Hospital in Khartoum State. When there was a positive confirmation of *P. aeruginosa*, the study supervisor went and collected the sample from the hospital. The samples were collected from patients suffering from urinary tract infections, respiratory tract infections, blood infections, and wound and ear infections. Data pertaining to the site of infection was collected from hospital records. The bacteria were preserved in 20% glycerol and peptone water and stored at -20°C

Phenotypic identity of the isolates was confirmed through conventional bacterial identification methods, such as Gram stain, oxidase test, and reactions in media containing sugars, such as Kligler Iron Agar, urease test and, citrate test. Pigment production was assessed using Muller Hinton agar, and then phenotypic identity confirmed by genotypic characterization using multiplex PCR, as previously described ^1^.

### Antimicrobial susceptibility testing

Muller Hinton medium (HiMedia, India) was prepared and sterilized as instructed by the manufacturer. Antimicrobial susceptibility testing was performed following the modified Kirby-Bauer disc diffusion method ^1^ and the results interpreted according to Clinical Laboratory Standards Institute guidelines (CLSI, 2007). The following antimicrobial discs (HiMedia, India) were used for sensitivity testing: Amoxicillin (25 µg), Cefotaxime (30 µg), Amoxicillin-Clavulanic acid (30 µg), Gentamicin (10 µg), Ciprofloxacin (5 µg), Chloramphenicol (30 µg) and Imipenem (10 µg). *P. aeruginosa* ATCC 27853 was used as quality control strain to control the performance of the test and ensure that the test is properly performed.

Phenotypic identity of the isolates was confirmed through conventional bacterial identification methods such as Gram stain, oxidase test, reactions in media containing sugars such as Kligler Iron Agar, urease test and citrate test.

### Genotypic analysis of bacterial isolates

Genomic DNA was extracted by simple boiling method ^18^. The extracted DNA was used as a template for amplification of target genes using multiplex PCR using TECHNE TC-312 (UK) thermocycler. Firstly, oprI and oprL primers ( Table 1) (Macrogen, Korea) were used to confirm the identification of *P. aeruginosa*. Seven primer pairs ( Table 1) (Macrogen, Korea) were used for detection of β-lactamases genes (bla _TEM_, bla _SHV_, bla _CTXM-1_, bla _VEB_, bla _OXA-1_) and class C genes (bla _AmpC_ and bla _DHA_). The oprI and oprL reaction was carried out with the following cycling conditions: denaturation at 95°C for 5 min; 33 cycles of denaturation at 95°C for 30 secs, annealing at 58°C for 30 sec and extension at 72°C for 30 secs, and final extension at 72°C for 5 min ^28^.

**Table 1.** Primer sequences.

Target gene	Primers	Sequences (5'-3')	Product size (bp)	References
*oprI*	F	ATGAACAACGTTCTGAAATTC	250	29
	R	CTTGCGGCTGGCTTTTTCCAG		
*oprL*	F	ATGGAAATGCTGAAATTCGGC	500	29
	R	CTTCTTCAGCTCGACGCGACG		
*TEM*	F	ATGAGTATTCAACATTTCCGTG	861	30
	R	TTACCAATGCTTAATCAGTGAG		
*SHV*	F	TTTATGGCGTTACCTTTGACC	1050	30
	R	ATTTGTCGCTCTTTACTCGC		
*CTXM-1*	F	GACGATGTCACTGGCTGAGC	499	31
	R	AGCCGCCGACGCTAATACA		
*AmpC*	F	ATCAAAACTGGCAGCCG	550	32
	R	GAGCCCGTTTTATGGACCCA		
*DHA*	F	AACTTTCACAGGTGTGCTGGGT	405	33
	R	CCGTACGCATACTGGCTTTGC		
*VEB*	F	CATTCCCGATGCAAAGCGT	648	34
	R	CGAAGTTTCTTTGGACTCTG		
*OXA-1*	F	GGCACCAGATTCAACTTTCAAG	564	35
	R	GACCCCAAGTTTCCTGTAAGTG		

β-lactamases detection was done in two batches; the first batch was used for detection of bla _TEM_, bla _SHV_, bla _CTXM-1_, bla _AmpC_ and bla _DHA_, while the second batch was used for detection of bla _VEB_ and bla _OXA-1_ genes. The first batch detection was done in 20 µl of final reaction mixture using Maxime PCR Premix kits (iNtRON Biotechnology, Korea), containing 13 µl of double distilled water (DDW), 0.3µl of each five forward and 0.3 µl of each five reverse primers (1.5 µl), 2 µl of Dimethyl sulfoxide (DMSO) and 2 µl of template DNA.

Amplification of the second batch was done in 20µl using Maxime PCR Premix kits (iNtRON Biotechnology, Korea), containing 14.4 µl of DDW, 0.4 µl of each two forward and 0.4 µl of each two reverse primers (0.8 µl), 2 µl of DMSO and 2 µl of template DNA. Cycling conditions for both amplification reactions were as follows: initial denaturation at 94°C for 2 minutes, then 35 cycles of denaturation at 94°C for 30 secs, annealing at 54°C for 30 secs and extension at 72°C for 50 secs, and final extension at 72° for 5 minutes.

Amplified products were analyzed by electrophoreses at 80 volts for 20 minutes on 1.5% agarose gel containing ethidium bromide and then visualized using UV transilluminator (Uvitec–UK) with 50bp or 100bp molecular DNA ladder (iNtRON Biotechnology, Korea).

### Data analysis

Statistical analysis of the data was performed using chi-square test (level of significance was 0.05) with SPSS software version 20 and GraphPad prism 5 demo.

## Results

From 121 clinical isolates collected from different hospitals in Khartoum State, only 80 (66%) were confirmed as *P. aeruginosa* through conventional methods and species-specific primers; the remaining 41 (34%) isolates were considered as other Gram-negative rod bacteria. The distribution of clinical isolates according to site of infection was as follows: urine, 34 (42%); wound swab, 24 (30%); ear swab, 8 (10%); sputum, 8 (10%); and blood, 6 (7.5%).

The results of antimicrobial susceptibility test for selected antibiotics are presented in Table 2.

**Table 2.** Antimicrobial susceptibility pattern of *Pseudomonas aeruginosa* (n=80).

Antimicrobial agent	Susceptible isolates N (%)	Resistant isolates N (%)
Ciprofloxacin	57 (71.2)	23 (28.8)
Gentamicin	61 (76.2)	19 (23.8)
Imipenem	72 (90)	8 (10.0)
Chloramphenicol	9 (11.2)	71 (88.8)
Amoxicillin/clavulanate	0	80 (100)
Amoxicillin	0	80 (100)
Ceftazidime	34 (42.5)	46 (57.5)
Cefotaxime	9 (11.2)	71 (88.8)

**Figure 1.**
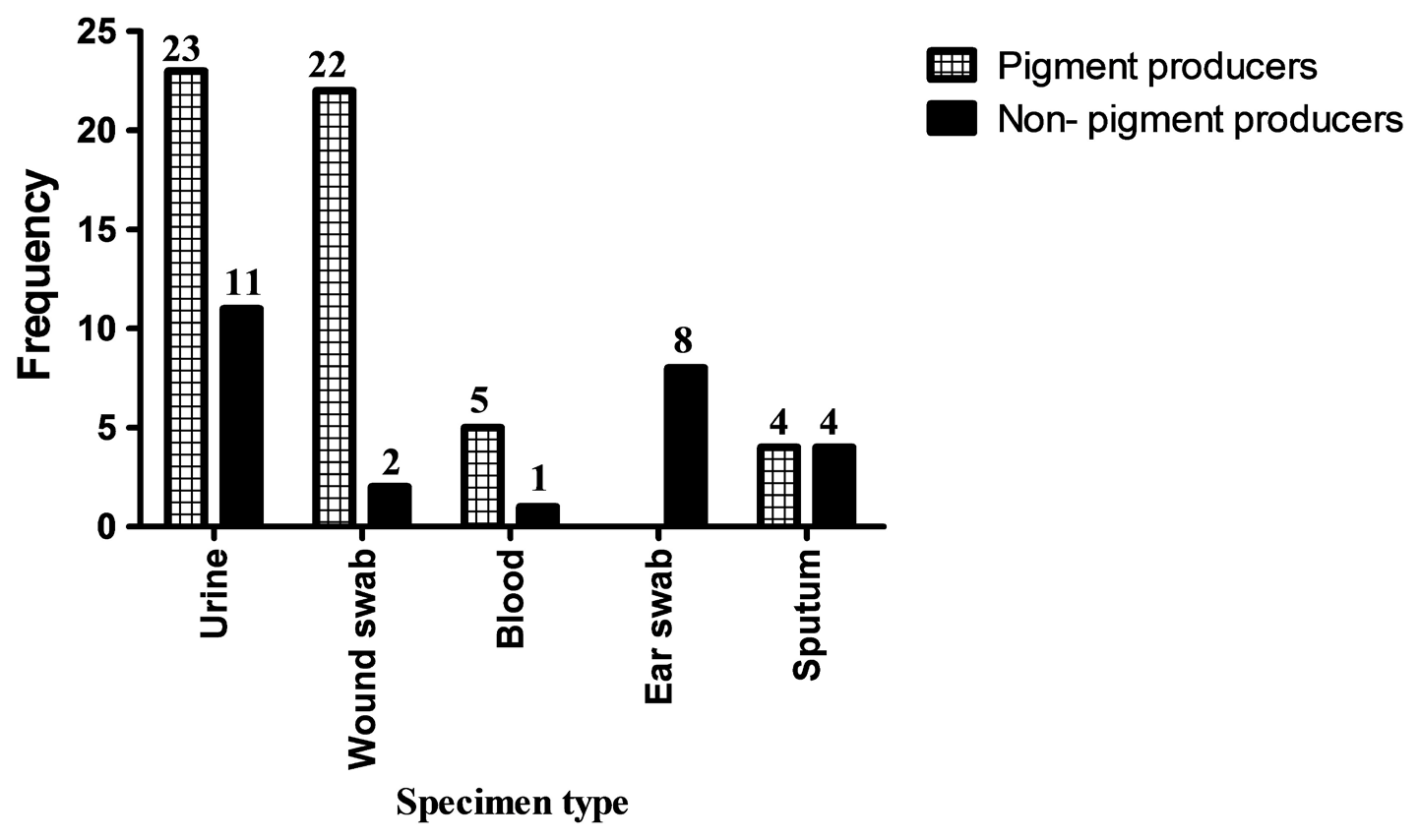
Frequency of *Pseudomonas aeruginosa* pigment producing isolates according to site of infection.

**Figure 2.**
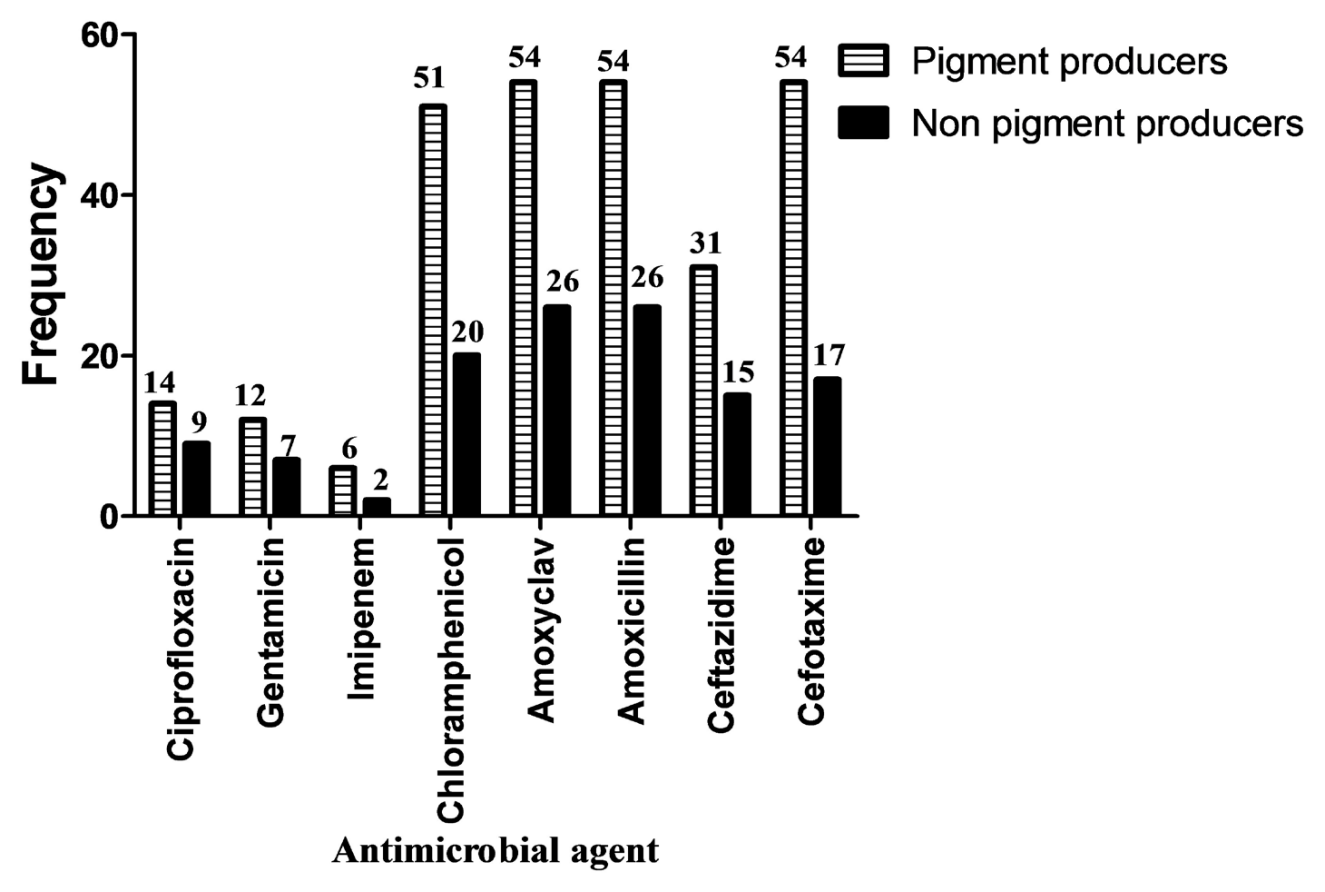
Frequency of *Pseudomonas aeruginosa* pigment producing isolates according to antimicrobial resistance.

Out of 80 *P. aeruginosa* isolates, 54 (68%) were Pyocyanin pigment producers, while 26 (32%) were not pigment producers. There was a significant association between pyocyanin pigment production and site of infection ( *P*=0.000) ( Figure 1). There was also a significant association between pigment production and resistance to Chloramphenicol ( *P*=0.020) and Cefotaxime ( *P*=0.000), while there was insignificant association between pigment production and resistance to other antimicrobials used in this study ( Figure 2).

**Figure 3.**
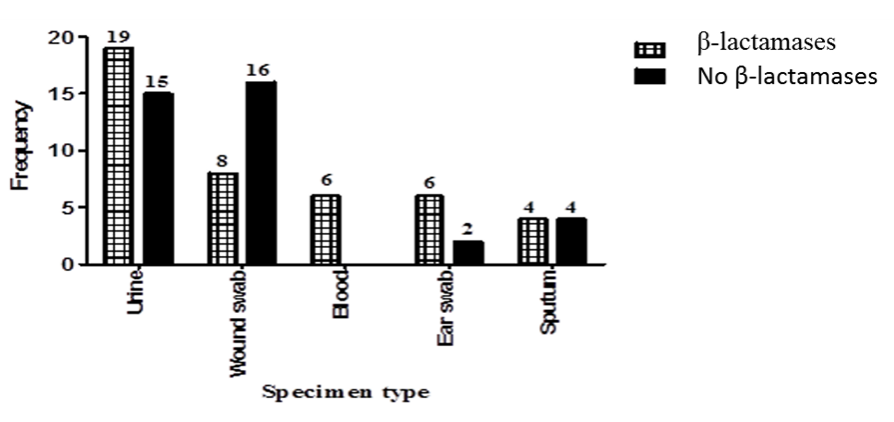
Frequency of β-lactamases ( *bla _TEM_, bla _SHV_, bla _CTXM-1_, bla _VEB_ or bla _OXA-1_*) among *Pseudomonas aeruginosa* isolates site of infection.

Molecular detection of β-lactamases showed that 43 (54%) of the isolates were positive for at least one β-lactamases gene, while 37 (46%) were negative for all genes ( Figure 3). The frequency of each gene presence among *P. aeruginosa* was as follows: bla _TEM_, 19 (44.2%); bla _SHV_, 16 (37.2%); bla _CTX-M1_, 10 (23.3%); bla _VEB_, 14 (32.6%); and bla _OXA-1_, 7 (16.3%). There was a significant association between the presence of *bla _TEM_, bla _SHV_, bla _CTXM-1_, bla _VEB_ or bla _OXA-1_* genes in *P. aeruginosa* and site of infection ( *P*=0.030) ( Figure 3). Co-presence of more than one of *bla _TEM_, bla _SHV_, bla _CTXM-1_, bla _VEB_ or bla _OXA-1_* gene among *P. aeruginosa* clinical isolates is presented in Table 3.

**Table 3.** Number of *Pseudomonas aeruginosa* isolates exhibiting co-presence of *bla _TEM_, bla _SHV_, bla _CTXM-1_, bla _VEB_, bla _OXA-1_* β-lactamases genes.

	*bla _TEM_*	*bla _SHV_*	*bla _CTXM-1_*	*bla _VEB_*	*bla _OXA-1_*
***bla***_*TEM*_	7	6	5	3	1
***bla***_SHV_	6	4	4	4	1
***bla***_CTXM-1_	5	4	0	1	1
***bla***_VEB_	3	4	1	3	3
***bla***_OXA-1_	1	1	1	3	2

**Figure 4.**
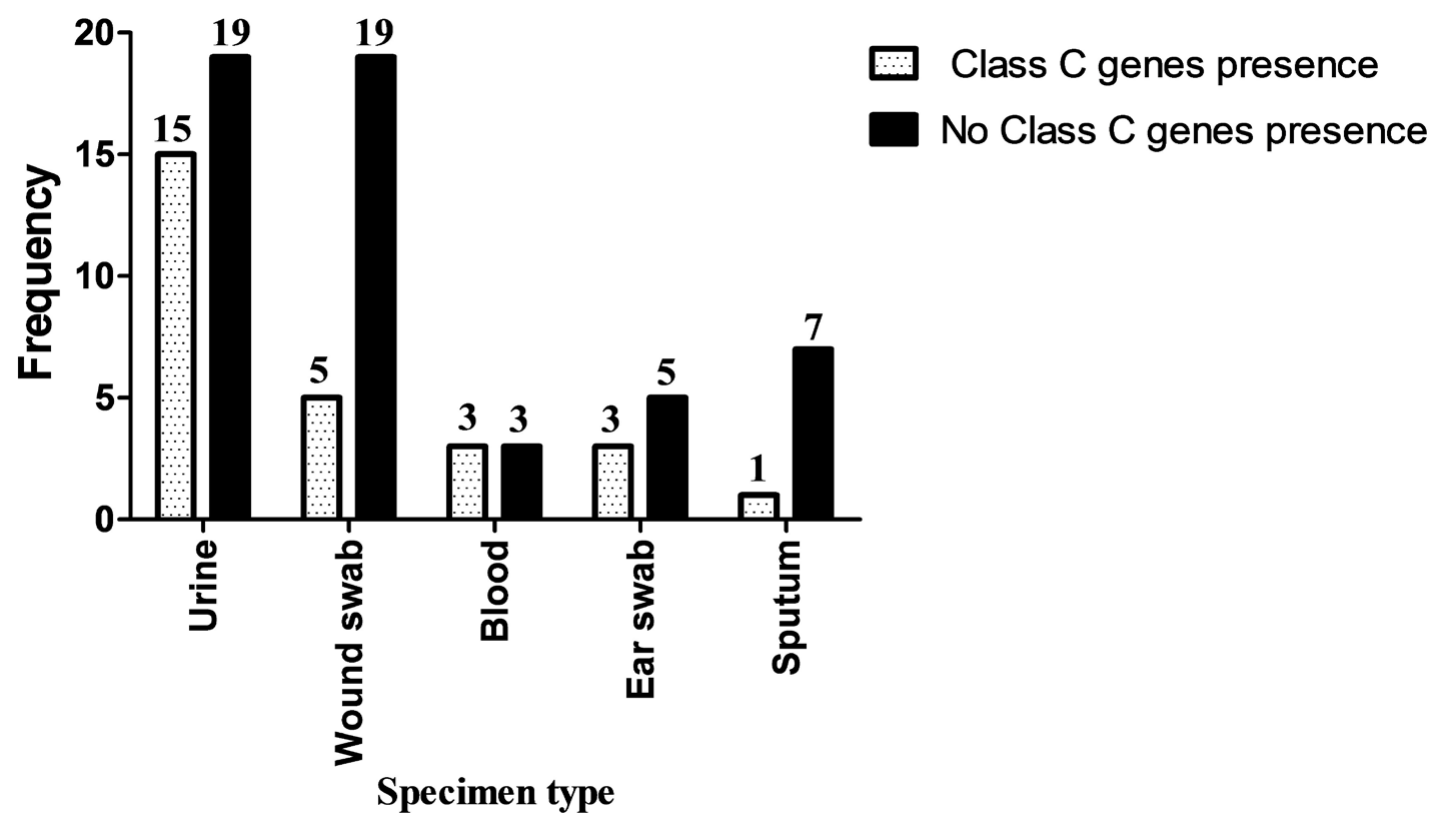
Frequency of class C genes among *Pseudomonas aeruginosa* isolates and site of infection.

Class C β-lactamases gene were positive in 27 (34%) *P. aeruginosa* isolates; while 53 (66%) were negative ( Figure 4). The frequency of class C β-lactamases genes was as follows: bla _AmpC_, 22 (81.5%); bla _DHA_, 8 (29.6%); and 3 (11.1%) isolates were positive for both genes. Association between presence of Class C β-lactamases genes and site of infection was insignificant ( *P*=0.215) ( Figure 4).

**Figure 5.**
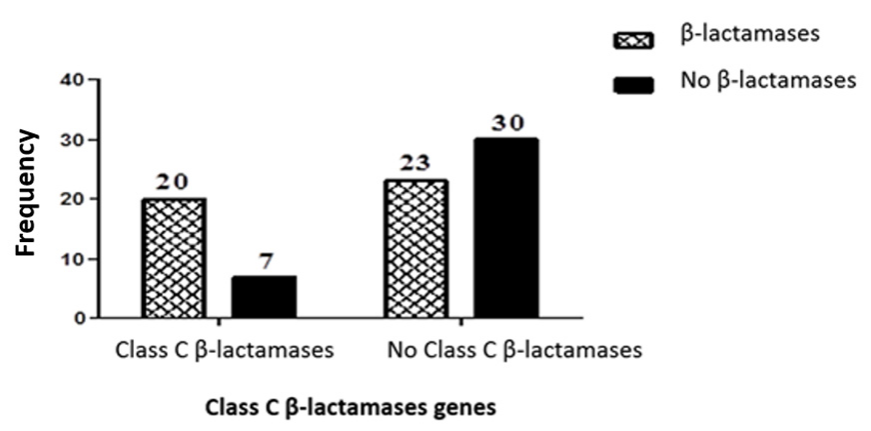
Frequency of β-lactamases ( *bla _TEM_, bla _SHV_, bla _CTXM-1_, bla _VEB_, bla _OXA-1_*) and class C β-lactamase genes among *Pseudomonas aeruginosa* isolates.

In total, 25% of *P. aeruginosa* isolates were positive for both β-lactamase ( *bla _TEM_, bla _SHV_, bla _CTXM-1_, bla _VEB_ or bla _OXA-1_*) and class C β-lactamase genes ( Figure 5). Co-presence of β-lactamase genes and class C β-lactamase among *P. aeruginosa* clinical isolates is presented in Table 4.

**Table 4.** Number of *P. aeruginosa* isolates exhibiting β-lactamases ( *bla _TEM_, bla _SHV_, bla _CTXM-1_, bla _VEB_, bla _OXA-1_*) and class C genes.

Number of genes	β-lactamases n (%)	Class C N (%)	Both classes N (%)	Total
1	16 (73)	6 (27)	-	22
2	7 (41)	1 (16)	9 (53)	17
3	**-**	**-**	6 (100)	6
4	**-**	**-**	5 (100)	5
5	**-**	**-**	1 (100)	1
6	**-**	**-**	**-**	0
7	**-**	**-**	**-**	0

Number and percentage of genes per isolate*

## Discussion

*Pseudomonas aeruginosa* is one of the main causative agents of serious nosocomial infections with increased reports of β-lactams resistant strains that makes treatment difficult and complicated ^^19^^. Production of β-lactamases is one of the most common mechanisms of β-lactam resistance ^^36^^.

In this study the frequency of β-lactamases genes among *P. aeruginosa* isolates was 53.8%. This frequency is close to a report from India by Qureshi and Bhatnagar (2016) ^^37^^, where the frequency of β-lactamases in *P. aeruginosa* isolates was 46%. However, the results of this study disagree with another report from India by Gupta *et al*. (2016) ^38^ where the frequency of β-lactamases in *P. aeruginosa* isolates was 22.9%. These discrepancies could be due to differences in strains of the clinical isolates, the antibiotics used or sample size.

The most abundant gene among the β-lactamases ( *bla _TEM_, bla _SHV_, bla _CTXM-1_, bla _VEB_, bla _OXA-1_*) producing *P. aeruginosa* isolates detected in this study was *bla*_TEM_ gene (19, 44.2%), followed by *bla*_SHV_, *bla*_VEB_, *bla _CTXM_*_-1_ and *bla*_OXA-1_ (16 (37.2%), 14 (32.6%), 10 (23.3%) and 7 (16.3%), respectively). Similar results were reported by Salah *et al*. (2016) ^39^ in Egypt concerning the presence of *bla*_TEM_, *bla*_SHV_ and *bla*_OXA-1_ (50%, 33% and 17%, respectively). The frequency of *bla*_TEM_ gene is also similar to that reported by Rafiee *et al*. (2014) ^40^, which was present in 39.2% of isolates. In a study in Iran by Sales *et al*. (2017) ^41^, similar results were reported concerning the presence of *bla*_CTXM-1_ (27.3%), while in India Jamali *et al*. (2017) ^15^ reported a higher frequency (57.5%) of this gene.

On the other hand, the frequency of *bla*_VEB_ and *bla*_SHV_ genes in this study differs from that reported in Iran by Bokaeian *et al*. (2014) ^42^ where *bla*_VEB_ gene frequency was 13.3%, while the frequency of *bla*_SHV_ gene was 6.6%. The report by Jamali *et al*. (2017) ^15^ concerning the genes *bla*_TEM_ (15%) and *bla*_SHV_ (75%) are also different from those reported in this study, and this could be due to the variation in strains of clinical isolates and the sample size used.

In this study, the frequency of class C β-lactamase genes in *P. aeruginosa* isolates was 27 (34%). This result is close to a report from India by Gupta *et al*. (2016) ^38^ where 43% of *P. aeruginosa* were AmpC producers, and disagrees with a report from Thailand by Katvoravutthichai *et al*. (2016) ^43^ where 11% of *P. aeruginosa* isolates were AmpC producers. In this study, out of the class C β- lactamase producing *P. aeruginosa* isolates, 22 (81.5%) and 8 (29.6%) isolates were positive for *bla*_AmpC_ and *bla*_DHA_ genes, respectively. Qureshi and Bhatnagar (2016) ^37^ in India reported that no *P. aeruginosa* isolates were positive for *bla*_AmpC_ gene, while Rafiee *et al*. (2014) ^40^ in Iran reported that 60.8% of *P. aeruginosa* were positive for *bla*_AmpC_ gene.

All *P. aeruginosa* clinical isolates tested in our study were resistant to Amoxicillin and Amoxicillin/clavulanate and this may be due to the misuse of antibiotics in Sudan ^44^, where plenty of antimicrobial agents are sold over the counter. This rate of resistance is higher than the rate of resistance reported by Ahmad *et al*. (2016) ^45^ in Pakistan where the resistance to Amoxicillin and Amoxicillin/clavulanate was 73.4% and 67.7% respectively, and this could be justified by the time difference between the studies, as well as the difference in the strains and antibiotics used.

The resistance rate of *P. aeruginosa* to Imipenem was 10% (n=8). This may be due to the infrequent use of Imipenem antibiotics. This percentage agrees with a study reported in Pakistan by Ahmad *et al*. (2016) ^45^ where 11.1% of *P. aeruginosa* isolates were resistant to Imipenem, while in Sudan Altom and Ahmed (2015) ^46^ reported that 5.7% *P. aeruginosa* isolates were resistant to Imipenem. This finding may indicate that carbapenem resistance is on the rise in *P. aeruginosa* isolates from Sudan.

In this study, the number of *P. aeruginosa* isolates resistant to Cefotaxime, Chloramphenicol and Ceftazidime was 71 (88.8%), 71 (88.8%) and 46 (57.5%), respectively. The rate of resistance to Cefotaxime in this study is different from that reported in Pakistan by Ahmad *et al.* (2016) ^45^ who found that 20.3% of *P. aeruginosa* isolates were resistant to Ceftazidime. The resistance rate in this study also disagrees with that reported by Albadawi (2010) ^47^ in Sudan who found that resistance of *P. aeruginosa* to Ceftazidime and Cefotaxime were 31% and 42%, respectively. These findings also indicate the rapidly increasing rates of *P. aeruginosa* resistance to antimicrobial agents in Sudan perhaps due to antibiotic misuse.

In this study, 23 (28.8%) and 19 (23.8%) of *P. aeruginosa* clinical isolates were resistant to Ciprofloxacin and Gentamicin, respectively. Altom and Ahmed (2015) ^46^ in Sudan also reported that 18.6% of *P. aeruginosa* were resistant to Gentamicin. Different results were reported by Ahmad *et al*. (2016) ^45^, where the percentage of resistance to Gentamicin and Ciprofloxacin were 74.3% and 44%, respectively. These percentages are much higher than those reported in this study probably due to their different geographical location, study time difference and the antibiotic-use rates.

In this study, there was significant association between β-lactamases production and site of infection ( *P*=0.030). *P. aeruginosa* isolated from blood showed the highest production of *bla _TEM_, bla _SHV_, bla _CTXM-1_, bla _VEB_ and bla _OXA-1_* genes followed by ear swab, urine, sputum and wound swab. This result agrees with a study in India reported by Basak *et al*. (2012) ^48^ where the highest β-lactamases producing *P. aeruginosa* isolates were from blood, but disagrees with Azizi *et al*. (2015) ^49^ in Iran who found that highest production was in *P. aeruginosa* isolated from wound followed by urine, sputum and blood. There was insignificant association between the presence of class C β-lactamase genes and site of infection ( *P*=0.215) found in the present study. The highest frequency of class C β **-**lactamases genes in *P. aeruginosa* were isolated from blood followed by urine, ear swab, wound swab and sputum. These results agree with the study in India reported by Basak *et al*. (2012) ^48^ in that the highest percentage of class C β-lactamase genes in *P. aeruginosa* were found in blood.

In the present study, there were four clinical isolates phenotypically sensitive to third generation cephalosporins (Ceftazidime and Cefotaxime) and genotypically positive for β-lactamases genes. This result indicates that *P. aeruginosa* may carry hidden unexpressed genes that could be detected through molecular techniques. This result agrees with a study in India reported by Bajpai *et al*. (2017) ^50^ where out of 38 phenotypically β-lactamases-negative isolates, 20 isolates were positive for β-lactamases genes.

In this study, the frequency of pigment producing *P. aeruginosa* isolates was 67.5%. In a study in India a higher percentage was reported where 82.5% of *P. aeruginosa* were pigment producers (Finlayson and Brown, 2011) ^51^. The present study revealed that there was no relationship between pigment production and pattern of antimicrobial resistance, except in Chloramphenicol ( *P*=0.02) and Cefotaxime ( *P*=0.00).

In this study, the relationship between pigment production and site of infection was significant ( *P*=0.000). *P. aeruginosa* isolated from wound infections were the highest pigment producing isolates followed by isolates from blood, urine and sputum. There was no pigment production in *P. aeruginosa* isolated from ear infections in this study.

The variations between the results of this study and other reports could be attributed to the difference in antibiotic usage patterns in each region, economical causes, geographical differences, sample size, differences in time in which the studies were performed, and study population. Despite the significance of the present study, there were limitations that should be avoided in future studies, such as the small sample size, phenotypic detection of β-lactamases, coverage of other β-lactamase classes, and gene sequencing should be done in order to confirm and to identify all the genes that are carried by *P. aeruginosa* strains in Sudan.

## Conclusion

This is study is of great importance as it raises attention to the existing problem of resistance to β-lactams in *P. aeruginosa* in Sudan. This study confirms the reports that a number of antibiotics are becoming useless for treating this problematic bacterium, since all the strains of *P. aeruginosa* isolates in this study were resistant to Amoxicillin and Amoxicillin/clavulanate. The best antibiotic sensitivity results obtained in this study were those of Imipenem, followed by Gentamicin and Ciprofloxacin. Moreover, *P. aeruginosa* isolates showed an increased rate of β-lactamase production with co-resistance with other classes of antibiotics. Of interest is the finding that clinical isolates were resistant phenotypically in high frequencies to Amoxicillin, Amoxicillin/clavulanate and a third generation antibiotic, Cephalosporin, and showed negative results genotypically, indicating that resistance to this family of antibiotics also exist by resistance mechanisms other than β-lactamases production. Also, our PCR results revealed that *P. aeruginosa* possesses hidden β-lactamases genes that can’t be detected phenotypically. Finally, this study highlighted for the first time the problem of misidentification of *P. aeruginosa* and other microorganisms in Khartoum hospitals as only 80 out of the 120 alleged isolates were confirmed to be *P. aeruginosa* through PCR.

## Data availability

### Underlying data

Figshare: SPSS, https://doi.org/10.6084/m9.figshare.12453287.v2 ^51^

This project contains the following underlying data:

- SPSS. sav (Result sheet for beta-lactamases detection and sensitivity testing)
- Data Dictionary. docx

Data are available under the terms of the Creative Commons Attribution 4.0 International license (CC-BY 4.0).
