# Supplementary material for: β-lactamases (bla TEM, bla SHV, bla CTXM-1, bla VEB, bla OXA-1) and class C β-lactamases gene frequency in Pseudomonas aeruginosa isolated from various clinical specimens in Khartoum State, Sudan: a cross sectional study
Source: F1000Res. 2021 Apr 1;9:774. Originally published 2020 Jul 27. [Version 3] doi: 10.12688/f1000research.24818.3 (PMC7737708; doi:10.12688/f1000research.24818.3)
Supplement: Peer review history

# Peer review: Reviewer response for version 2

**DOI:** 10.5256/f1000research.29444.r74648

**Published:** 2020-12-14

**Authors:** Muhammad Qasim

**Affiliations:** 1 Department of Microbiology, Kohat University of Science and Technology, Kohat, Pakistan

The manuscript “Extended spectrum β-lactamases and class C β-lactamases gene frequency in  Pseudomonas aeruginosa isolated from various clinical specimens in Khartoum State, Sudan: a cross sectional study” describe important findings on drug resistance (ESBL and Class C Beta lactamases) of *P. aeruginosa* in Khartoum State, Sudan. The author claimed the study to be the first one to describe the exact frequency of ESBL producing *P. aeruginosa* in Khartoum, Sudan.

 On the whole the research is scientifically carried out and written. Following are few minor suggestions

- In the abstract section, the author has mentioned that 40 isolates were other bacterial species. I think the objective of the study is the molecular detection of beta lactamases in *P.aeruginosa* so there is no need mention about other bacterial species.
- The statement “121 Ps. aeruginosa clinical isolates from various clinical specimens were used in this cross-sectional study conducted in Khartoum State. A total of 80 isolates were confirmed as *Ps. aeruginosa* through conventional identification methods and species specific primers” is really confusing. If 80 *P. aeruginosa* isolates were finally analyzed for drug resistance then there no need to state 121 *P. aeruginosa* clinical isolates.
- As mentioned above the focus of manuscript is drug resistance in *P. aeruginosa*, so the irrelevant information such as Pyocyanin pigment production should be deleted.
- Though in methodology section, the author has claimed statistical tests (chi square, p value), but there is no information on statistical tests in figures. I think statistical values should be included in figures.
- In all tables, please proper define the abbreviations, number and percentage (n(%)) in foot note.
- Figures should be properly labeled (Frequency (n)).
- The percentage should be added in all tables, as it will help the readers to understand and interpret the tables in better way.
- Some of the references are inconsistent/incomplete which should be arranged as per journal format.

Is the work clearly and accurately presented and does it cite the current literature?

Yes

If applicable, is the statistical analysis and its interpretation appropriate?

Partly

Are all the source data underlying the results available to ensure full reproducibility?

Yes

Is the study design appropriate and is the work technically sound?

Yes

Are the conclusions drawn adequately supported by the results?

Yes

Are sufficient details of methods and analysis provided to allow replication by others?

Yes

Reviewer Expertise:

Medical Microbiology

I confirm that I have read this submission and believe that I have an appropriate level of expertise to confirm that it is of an acceptable scientific standard.

# Response

**Published:** 2020-12-19

**Authors:** Dina Naser

**Affiliations:** Central Laboratory, Sudan

Thank you for these comments, minor changes have been done following the reviewer’s comments. Changes include specifying the final included sample size, addition of results percentages to the tables with minor edits in the figures titles and in the abstract the Beta-lactamases genes naming changed according to the title.

# Peer review: Reviewer response for version 2

**DOI:** 10.5256/f1000research.29444.r71387

**Published:** 2020-09-22

**Authors:** Nobumichi Kobayashi

**Affiliations:** 1 Department of Hygiene, Sapporo Medical University School of Medicine, Sapporo, Japan

The revised version is described appropriately using correct scientific terms.

Is the work clearly and accurately presented and does it cite the current literature?

Partly

If applicable, is the statistical analysis and its interpretation appropriate?

Partly

Are all the source data underlying the results available to ensure full reproducibility?

Partly

Is the study design appropriate and is the work technically sound?

Partly

Are the conclusions drawn adequately supported by the results?

Partly

Are sufficient details of methods and analysis provided to allow replication by others?

Yes

Reviewer Expertise:

microbiology, public health

I confirm that I have read this submission and believe that I have an appropriate level of expertise to confirm that it is of an acceptable scientific standard.

# Peer review: Reviewer response for version 1

**DOI:** 10.5256/f1000research.27382.r68073

**Published:** 2020-08-26

**Authors:** Nobumichi Kobayashi

**Affiliations:** 1 Department of Hygiene, Sapporo Medical University School of Medicine, Sapporo, Japan

This is a kind of primitive study that provides only limited information. Only importance is that this study was done in Sudan. However, still number of isolates were low, and errors in descriptions of technical terms are found in this manuscript.

1. Abstract: Number of *P. aeruginosa* should be written as "80". "121" should not appear here, because 41 isolates were not *P. aeruginosa*.
2. " *Ps. aeruginosa*" should be corrected as " *P. aeruginosa*", as formal abbreviation.
3. Amoxyclav may be a commercial name of the drug. It should be written as general name. Maybe it is "amoxicillin/clavulanate". 
4. Authors may have serious misunderstanding regarding the term of ESBL. NOT all of TEM, SHV, OXA type beta-lactamases are ESBL. TEM-1, 2, SHV-1, OXA-1 are not ESBL. Other subtypes of TEM, SHV, and OXA are called as "ESBL". Those subtypes can be identified only by sequencing of full-length ORF of these genes. In this manuscript, only CTX-M type gene can be called as "ESBL". However, it is not sure that TEM, SHV, OXA type genes are ESBL genes or not, because sequence was not determined. In this regard, this manuscript must be revised substantially. Authors can mention CTX-M as ESBL, others should not be referred as ESBL, but just individual enzyme nams, e.g., "TEM gene". According to this alteration, whole manuscript should be rewitten.
5. CTX-M-1 should be written as "CTX-M-1 group". CTX-M types cannot be determined by only PCR. Authors should read and study literature on ESBL and betalactamases of bacteria, to exactly understand their nomenclature and definition.
6. Table 3 cannot be understood. Authors should make a table to show combination pattern of beta-lactamases and their frequency.
7. Figure 5: "Class AmpC genes" is wrong. It should be "Class C beta-lactamase" or "AmpC gene". In this case, "AmpC gene" is better.

Is the work clearly and accurately presented and does it cite the current literature?

Partly

If applicable, is the statistical analysis and its interpretation appropriate?

Partly

Are all the source data underlying the results available to ensure full reproducibility?

Partly

Is the study design appropriate and is the work technically sound?

Partly

Are the conclusions drawn adequately supported by the results?

Partly

Are sufficient details of methods and analysis provided to allow replication by others?

Yes

Reviewer Expertise:

microbiology, public health

I confirm that I have read this submission and believe that I have an appropriate level of expertise to state that I do not consider it to be of an acceptable scientific standard, for reasons outlined above.

# Response

**Published:** 2020-09-06

**Authors:** Dina Naser

**Affiliations:** Central Laboratory, Sudan

-  Classification of ***bla _TEM_, bla _SHV_, bla _CTXM-1_, bla _VEB_***and ***bla _OXA-1_*** changed from ESBLs to β-lactamases enzymes generally, they named in the draft as individual genes and classified as **β** **-lactamases **group.

This change applied in the whole draft including tables and figures, even the title changed to **“** **β-lactamases** ***(bla _TEM_, bla _SHV_, bla _CTXM-1_, bla _VEB_, bla _OXA-1_*** **)** **and class C β-lactamases gene frequency in *Pseudomonas aeruginosa* isolated from various clinical specimens in Khartoum State, Sudan: a cross sectional study”**

- In abstract: The sample size mentioned as the confirmed **80** *P. aeruginosa* isolates, the remaining **41** isolate other than *P. aeruginosa *deleted.
- All “ ***Ps. aeruginosa*** *” *in the text *c*hanged to “ ***P. aeruginosa*** *” *as formal abbreviation.
- All “ **Amoxyclav**” in the text changed to “ **amoxicillin/clavulanate**”
-  " **Class AmpC genes**" in figure 5 changed to “ **Class C** **β** **-lactamase**”
- “ **ESBLs**” changed to “ **β** **- lactamases**” in figure 3 and figure 5.
